# An Anecdotal Outpatient Approach to Caring for Patients With End-Stage Hematologic Malignancies

**Published:** 2018-03-01

**Authors:** Mark Honor

**Affiliations:** H. Lee Moffitt Cancer Center and Research Institute, Tampa, Florida

## Abstract

Historically, hospice has been a poor option for patients with end-stage hematologic malignancies, largely due to the need for regular transfusions to sustain life near the end, and to a lesser extent the treatment of curable emergent infections. In many cases, hospice is a viable and favorable option for patients with solid tumors who are out of treatment options yet have many months to live. For patients with hematologic malignancies with fewer than 6 months to live, although tens of transfusions may be required, they may have a relatively good quality of life when those transfusions are provided. I present a long-term approach to this unique population using an outpatient approach with transition to hospice. The needs of this population are markedly different from the published recommendations regarding patients with solid tumors who are either out of treatment options or have progressive disease.

I have been managing patients with hematologic malignancies for over 10 years at the H. Lee Moffitt Cancer Center in Tampa. I spent the first 7 years in the stem cell transplant department, and more than 3 years ago I started working at a satellite location to our main hospital in the capacity of a PA with responsibilities that included covering the side effects of infusion patients and evaluating medical oncology patients prior to chemotherapy.

There is a significant population of patients with hematologic malignancies who come regularly for lab checks. Within the overall group of these patients, there are several subgroups: those with an acute diagnosis on therapy, patients with stable disease on therapy (non-Hodgkin lymphoma, myeloma), those in remission for long-term observation, pre–bone marrow transplant (BMT) patients, post-BMT patients with relapse, and patients with progressive/end-stage disease. Although I manage all of these groups under the supervision of the hematologists, the latter two groups presented a particular problem: How do I get them blood and/or platelets in a timely fashion? Purely out of necessity, I developed an outpatient approach to this unique population in an ambulatory care center. The process is not meant to be a standardized approach, but more of an example of a process that works at our institution, with the least burden to the patients.

By Florida state law, blood products must be stored in a state-licensed blood bank. We solved the platelet issue easily. Each morning we have a courier deliver two separate bags of platelets to the facility, which are then stored appropriately and can be used for any patient since platelets do not (usually) need to be crossmatched. If I anticipate that more patients may need platelets on any given day, I can order more. If the units are not used, they are sent back to the blood bank with the last courier.

The bigger challenge was getting a type and crossmatch sample from the clinic via courier to the blood bank and then getting the blood delivered back to my facility in a timely fashion. Unfortunately, the minimum turnaround time for receipt of compatible units is 4 hours and then another 3 to 4 hours to transfuse the blood. Therefore, from lab appointment to transfusion completion, a patient’s day could be 8 or more hours. This is obviously not ideal. Due to the factors above, I had to come up with an algorithm of sorts that took into consideration appointment timing and the anticipated need for transfusion (see Table 1).

**Table 1:**
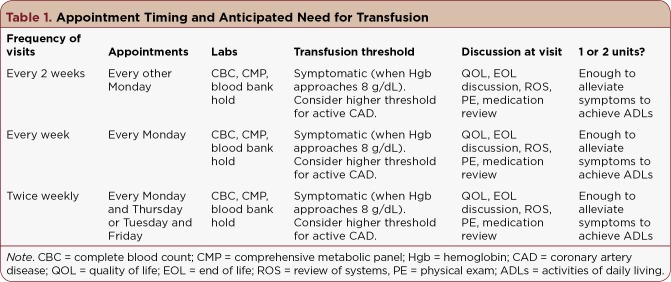
Appointment Timing and Anticipated Need for Transfusion

## TRANSFUSION GUIDELINES

National Comprehensive Cancer Network (NCCN) Guidelines state that the goal of transfusions is to prevent or treat the deficit of oxygen-carrying capacity in the blood. In symptomatic anemia (including tachycardia, tachypnea, and postural hypotension), the transfusion goal is to maintain hemoglobin as needed for the prevention of symptoms (NCCN, 2011). I would add that in the end-stage population, fatigue, dyspnea on exertion, and the inability to perform activities of daily living should be considered legitimate symptoms when considering transfusions. The American Society of Hematology Choosing Wisely recommendations are for inpatients and state, "Do not transfuse more than the minimum number of red blood cell (RBC) units necessary to relieve symptoms of anemia or to return a patient to a safe hemoglobin range (7–8 g/dL in stable, noncardiac, inpatients)" (Hicks et al., 2013; see Table 2).

**Table 2:**
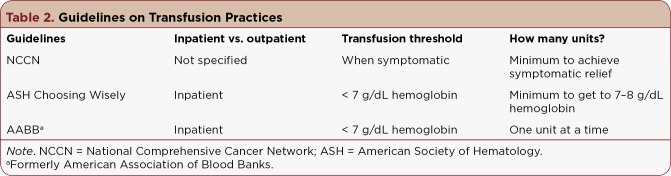
Guidelines on Transfusion Practices

The Clinical Practice Guidelines from the AABB recommend that there should be a restrictive RBC transfusion practice in which transfusion is not considered until the hemoglobin level is 7 g/dL for hospitalized adult patients who are hemodynamically stable (Carson et al., 2016). The 7 to 8 g/dL hemoglobin range for inpatients may be appropriate for most patients, but consider that inpatients are largely sequestered in their hospital rooms, are in an environment where their physical activity is limited, and are closely monitored. I would argue that most of my patients (all outpatients) who are trying to live a somewhat normal life cannot tolerate hemoglobin in the 7 to 8 g/dL range without being symptomatic.

## PERSONALIZED APPROACH

At what hemoglobin level do I transfuse? Do I transfuse one or two units at a time? I usually transfuse based on symptoms. I have a few patients who are very comfortable in the outpatient setting with a hemoglobin of 7.5 g/dL. I have others who are markedly symptomatic at a hemoglobin of 8.5 g/dL. I also have to consider how long they maintain their hemoglobin levels based on past lab trends. Initially, this trend can take 2 to 3 weeks to be established and is always subject to change in the case of progression of disease or the need for palliative chemotherapy (e.g., decitabine, 5-azacitidine, hydroxyurea). Once I figure out how long patients hold their hemoglobin, I can schedule them accordingly (see Table 1).

I transfuse platelets prophylactically when they are less than 10,000 µL, and if there is bleeding, below 50,000 µL (Szczepiorkowski & Dunbar, 2013). The Szczepiorkowski and Dunbar article in the American Society of Hematology transfusion guidelines addresses therapeutic and prophylactic transfusions (Szczepiorkowski & Dunbar, 2013). If a patient is already bleeding on presentation, the transfusion is considered therapeutic. If the patient is not bleeding, the transfusion would be considered prophylactic. I sometimes employ a prophylactic approach if the platelets are less than 20,000 µL heading into a "postchemo" weekend and I know they will be below 5,000 µL if I wait until the Monday visit based on the previous trend. If any of these patients have acute bleeding from thrombocytopenia or acute symptomatic anemia on a weekend, they often end up being admitted to outside/local hospitals because such facilities will not transfuse these patients in the ER and the patients end up hospitalized.

Such admissions have significant consequences to consider: breaking with continuity of care, cost to patient, cost to insurance company, risk of nosocomial infection, and patient unfamiliarity with outside hospital/doctors/process. I give each patient my email address to use during business hours and my cell number to use outside of office hours. Any outside hospitalization is antithetical to my goals with these patients. I would rather be aware of an emergency at 9:30 pm on a Friday night than find out about the issue on Monday morning. We also have a hematology/oncology fellow on call outside of clinic hours.

In our practice, I have found that a small percentage of physicians request that we (the primary oncology team) take over full care of these patients because of the complexity of the primary disease as well as the side effects of treatment. Necessity required me to embrace this new role. I am willing to manage any medical condition initially. If my therapy works, then I will manage it long term. If I find that a patient’s condition is more serious than I can handle (e.g., brittle diabetes mellitus), I will get a specialist involved. I frequently communicate with primary providers to establish patient status and goals.

It is recommended that erythropoietin-stimulating agents (ESAs) not be used in the treatment of anemia associated with malignancy in patients who are not receiving concurrent myelosuppressive chemotherapy. Use of ESAs in patients with lower-risk myelodysplastic syndrome to avoid transfusions is an exception to this recommendation (Rizzo et al., 2010). It has been my experience that the ESAs do not work in the end-stage population who have either bone marrow failure or progressive disease. I have had many low-grade myelodysplastic syndrome patients on these agents for months to years, but when the agents lose efficacy, I revert to transfusions.

## END-OF-LIFE CARE

By necessity, I have become adept at end-of-life discussions. Historically, most end-stage patients with hematologic malignancies die in the hospital (Howell et al., 2015; Sexauer et al., 2014). Again, this is antithetical to my goals for this population. Once I establish transfusion requirements and rapport, I will then discuss long-term goals with patients. Many of them ask, "How am I going to die?" I am honest to the best of my ability when answering this question. I will state that these particular patients I am asked to manage have either chosen not to receive aggressive therapy or are ineligible for such therapy. By the time I get to see them, there are no unreasonable expectations. The social worker ensures that each patient has advance directives, and I get a do-not-resuscitate (DNR) order signed for each patient.

This is also a good time to define palliative care vs. hospice care. Palliative care is meant to be a comprehensive approach to improving the quality of life for suffering patients. The first part of defining palliation is to make a contrast that cure is not the goal, but rather comfort. It is important to clearly outline the course of the illness and how the palliative care will be delivered. As mentioned previously, multiple transfusions are the biggest part of that care. Pain management, management of other medical conditions, psychosocial support, and family support are also part of that care. I will then discuss the goals of hospice, which are distinctly different in this population vs. the solid tumor population. I discuss with them that hospice is the better choice of care when the disease has progressed past the point where transfusions will help. During the end-of-life conversation I ask the patients if they want to die at home or elsewhere. Most people want to die at home, but some prefer a hospice house. It has been reported in the literature that some choose to die in the hospital, but I have never personally had a patient say to me that they preferred this option (Howell et al., 2015; Hui et al., 2014; Murray, 2011; O’Neill, 2015; Sexauer et al., 2014).

I will then recommend a hospice consult with the intent that this special population will likely not activate hospice until very close to the end. This is the exact opposite of the currently published guidelines of fewer than 6 months to live for solid tumor patients (Medicare.gov, 2017; NCCN, 2017). The intent is to get the patient familiarized with the hospice company and to get the paperwork out of the way. Throughout this process, the one area I have been challenged with is pain management. Based on Florida state law, PAs have not had the privilege to prescribe opioids until recently, but with the new privileges we may only prescribe 7 days at a time. This is not optimal for my patients; therefore, I request a supportive care consult for every patient with progressive/refractory disease or end-stage disease. The supportive care team manages pain and psychological well-being. They are also supportive of this prehospice model that I have created. They are experts at hospice and constructively reinforce the same discussion that I continually revisit with these patients (NCCN, 2017).

It is common for these patients to have an acute event and/or progression of disease. Urgent matters start with a call to nursing triage and escalate to a provider if they are truly urgent. A discussion regarding urgent vs. emergency issues should occur between patients and caregivers in advance. Urgent issues are seen the same day in clinic. Neutropenic fever, neurologic symptoms, and chest pain are considered emergencies. Regarding emergencies, if a DNR order is in place, the patient is treated supportively. If performance status rapidly declines, hospice is activated. If no DNR is in place, 911 is activated.

## CONCLUSIONS

I have been very fortunate to be supervised by an extremely supportive group of hematologists. They have given me the opportunity to mold a process that works very well for their practice, hospital utilization, and patient care. They are always available to me or the patient for consultation, but as the disease progresses, I see these patients almost exclusively as I care for them during end-stage progression and transition to hospice, with the ultimate goal of keeping them out of the hospital.

In the past 3 years I have cared for more than 30 patients using this approach. I have had a patient live as long as 18 months with this approach, and some others live just over a month. In my experience, most patients expire within 5 to 10 days of activating hospice. The needs of this population are markedly different from the published recommendations regarding patients with solid tumors who are either out of treatment options or have progressive disease (Sexauer et al., 2014). I have kept in touch with several of the family members of the patients I have taken care of. They all state that the care their family member received was compassionate and thoughtful and provided dignity in the face of an incurable terminal illness.
